# Do educational interventions reduce the gender gap in communication skills?- a systematic review

**DOI:** 10.1186/s12909-024-05773-9

**Published:** 2024-07-31

**Authors:** Alexis M. Driscoll, Rohan Suresh, George Popa, Leif Berglund, Amanda Azer, Helen Hed, Yajie Duan, Alice Chu, Aleksandra McGrath

**Affiliations:** 1 Department of Orthopaedic Surgery, Rutgers New Jersey Medical School, Newark, NJ 07103 USA; 2 Department of Clinical Sciences, Umeå university, Umeå, Sweden; 3 Umeå University Library, Umeå, Sweden; 4 Department of Statistics, Rutgers University, New Brunswick, NJ 08901 USA; 5 Department of Surgical and Perioperative Sciences, Umeå university, Umeå, Sweden

**Keywords:** Medical education, Gender, Communication skills, Medical students, Communication training

## Abstract

**Background:**

Efficient doctor-patient communication is essential for improving patient care. The impact of educational interventions on the communication skills of male and female students has not been systematically reviewed. The aim of this review is to identify interventions used to improve communication skills in medical curricula and investigate their effectiveness in improving the communication skills of male and female medical students.

**Methods:**

A systematic review of the literature was conducted using the PRISMA guidelines. Inclusion criteria were as follows: used intervention strategies aiming to improve communication skills, participants were medical students, and studies were primary research studies, systematic reviews, or meta-analyses.

**Results:**

2913 articles were identified based on search terms. After title, abstract, and full-text review, 58 studies were included with interventions consisting of Training or Drama Courses, Curriculum-Integrated, Patient Learning Courses, and Community-Based Learning Courses. 69% of articles reported improved communication skills for both genders equally, 28% for women more than men, and 3% for men more than women. 16 of the 58 articles reported numerical data regarding communication skills pre-and post-intervention. Analysis revealed that post-intervention scores are significantly greater than pre-intervention scores for both male (*p* < 0.001) and female students (*p* < 0.001). While the post-test scores of male students were significantly lower than that of female students (*p* = 0.01), there is no significant difference between genders for the benefits, or difference between post-intervention and pre-intervention scores (*p* = 0.15), suggesting that both genders benefited equally.

**Conclusion:**

Implementation of communication training into medical education leads to improvement in communication skills of medical students, irrespective of gender. No specific interventions benefitting male students have been identified from published literature, suggesting need of further studies to explore the phenomenon of gender gap in communication skills and how to minimize the differences between male and female students.

**Supplementary Information:**

The online version contains supplementary material available at 10.1186/s12909-024-05773-9.

## Background

Efficient doctor-patient communication is essential for improving patients’ satisfaction, compliance with treatment and outcomes [1, 2] Good communication skills have benefits for physicians, such as increase in job-satisfaction, well-being, and burnout prevention [3]. Despite the undisputed importance of communication skills, there is a well-documented gender gap between female and male physicians [4]. Regardless of the methods of assessment, both self-reported and observed communication skills and their components, such as empathy, patient-centered care, perspective-taking, and verbal and non-verbal communication skills show that male medical students score significantly lower than female medical students [5–11]. The differences persist after medical education and female physicians in general tend to be more patient-centered, and have more positive attitudes toward patient-centeredness, communication skills, and empathy than males [12].

Educational interventions to improve communication skills are nowadays part of medical programs globally [13]. Good communication skills are seen as one of the key competencies for medical graduates by both experts in curriculum design and students themselves [14]. Educational intervention exists as a broad term that encompasses many different formats. Different types of interventions include integrating a topic, such as development of communication skills, into an existing clinical curriculum or creating an entirely new specific course dedicated to this topic and adding it alongside a current curriculum. Integrated training and stand-alone training both aim to enhance students’ communicative ability, but both have benefits and drawbacks [15]. The effectiveness of interventions in medical education is often mapped against the Kirkpatrick hierarchy, from measuring the satisfaction of students with the intervention to evaluating organizational change and benefits to stakeholders (i.e. patients) [16]. While limited to tangible endpoints and omitting how medical students acquire competence in communication skills through practice and situated cognition, the Kirkpatrick hierarchy has been adapted by The Best Evidence Medical Education (BEME) Collaboration as part of an assessment of the quality of educational interventions [17].

The effectiveness of educational interventions aiming to improve communication skills in medical students has been investigated in a recent meta-analysis [18]. In general, the educational interventions were found to have positive effects compared to usual curricula on outcomes such as overall communication skills, empathy, and information gathering, however, the effect sizes were generally small [18]. There is no consensus on how effective stand-alone courses in communication skills are when compared to integrated communication skills teaching, even if both are appreciated by students themselves, given there are components of experiential learning [19].

Kirkpatrick’s model has served as the primary organizing design for training evaluation [17, 20]. This model comprises five essential levels of evaluation. The first level identifies participation in or completion of the intervention. The second level focuses on the participants’ perceptions of the course and their satisfaction. The third level is where participants acquire knowledge, skills, and changes in their attitudes and behaviors. The fourth level measures whether learned knowledge and attitudes result in changes in the workplace. The fifth level focuses on benefits to stakeholders, i.e. patients.

The impact of educational interventions in communication skills on male and female students has not been systematically reviewed and the most recent and comprehensive systematic Cochrane review provides only aggregated data for both genders [18].

Individualizing educational and learning paths is one of the challenges of modern education. There is a growing body of research showing the benefits of personalized, adaptive, and individualized learning in higher education, based on the students’ prior experiences, capabilities, and characteristics [21–23]. However, operationalizing these pathways is complex. Current literature in the field of medical education focuses heavily on personalized learning for struggling medical learners and technology assisted personalized learning, while studies investigating the learners’ needs are scarce [24–26]. In general, the concept of individualizing the learning pathway operates at the level of creating an adaptive learning environment at individual level, however in the aim of creating such environment, knowledge is needed of contributing factors operating at group level. This approach to generating knowledge needs to be exercised with care, as generalizing and stereotyping about gender might be detrimental to the educational process [27]. On the other hand, examining if the educational practices could be better tailored to all students could be strongly beneficial for patients as poor communication skills are implicated in the presence of recently discovered phenomenon of gender discordance between health provider and patient, where female patients of male physicians or surgeons are at higher risk of complication, death and worse health outcomes in general [28].

The aim of this review is to investigate the educational needs of male and female medical students through identification of the range and type of interventions, both didactic and experiential, aiming to improve communication skills as part of undergraduate medical curricula and to investigate how effective they are in improving skills of male and female medical students. In the light of differences in communication skills we hypothesize that there are gender differences in effectiveness of educational interventions. The conclusions of this study may highlight if any of the currently used educational interventions could be prioritized to improve the communication skills of male students to a higher extent and potentially narrow the gender gap.

## Materials and methods

### Protocol

A systematic review of the literature was conducted based on the PRISMA guidelines [29]. A protocol was registered with PROSPERO prior to the review (A.A).

### Eligibility criteria

Studies which met the following criteria were included:

- Intervention: strategies aiming to improve overall communication skills or components of communication skills (relationship building, eliciting patient perspective, active listening, using non-verbal communication, information gathering, increasing empathy, reaching consensus, sharing decisions, and providing closure).
- The participants: undergraduate medical students. If data for medical students could be separated from other participants, the study was included. Only studies that mentioned the participants’ gender and the number of participants in each gender were included.
- Types of studies: primary research studies, systematic reviews, meta-analyses, and conference abstracts.
- Assessment: assessment of effects of intervention for communication skills, across components suggested by Kalamazzo Consensus, including self-reported attitudes and assessment of observed behaviors during interactions with real or simulated patients, surveys of patients’ experience in clinical interactions, and examinations using oral, essay, or multiple-choice response questions [30]. For the study to be included in the subgroup analysis, these assessments needed to provide numerical outcomes.

Studies not fulfilling inclusion criteria were excluded. Additionally, when outcomes were considered, studies assessing outcomes up to the level II according to Kirkpatrick, ex. satisfaction with courses were excluded [17]. Any source of gray literature beyond conference abstracts and proceedings were excluded as they were unlikely to align with the objective of this review. There was no restriction on the publication date or the language of publication.

### Search strategy

The concepts method was used to build the search strategy, including concepts of ‘medical student’, ‘educational intervention’ ‘communication skills’ and ‘male/female/gender’. A scoping pilot search was conducted by the university librarian (H.H.) for the PubMed database. To assess the sensitivity, a file containing 10 key studies which fulfill the inclusion criteria was created and cross-checked with results of pilot search. Following this step, the strategy was refined and adapted to the remaining databases, with the search string adjusted to conform to the particulars of each database (Supplemental Table 1). Searches of Scopus/MEDLINE/Embase, CINAHL, Web of Science, Google Scholar, PsycINFO, and Eric were performed between the 28th of January and the 3rd of February 2022. In addition, a direct search of journals in the field of medical education was conducted using appropriate keywords (Medical Teacher, Medical Education, BMC Medical Education, Medical Education Online, The Clinical Teacher, Perspectives on Medical Education, Teaching and Learning in Medicine, and Journal of Surgical Education and Advances in Health Sciences Education). Reference lists of included studies were cross-checked.

### Study selection

The title and abstract screening and full text screening were done by one of the authors (A.D., R.S., G.P.), followed by independent sampling by another author. Disagreements during the screening process were resolved during reviewer team meetings with the senior authors (A.C. and A.M.).

### Data extraction

Data from eligible studies were extracted based on pre-selected criteria. Datapoints included Manuscript ID, authors, publication date, journal, study design, educational intervention, outcome measure, and reported results. Results included outcomes reported by the article and numerical values for assessment of outcomes. For a paper to have numerical outcomes sufficient for data analysis, it must have included numerical data stratified by gender, on a scale with which the maximum and minimum values were provided. Data were extracted (A.D., R.S., G.P.) and verified (L.B.). Queries were resolved (A.D.) in discussion with senior author (A.M).

### Data analysis and synthesis

Data were analyzed by intervention type and outcome measures. After the final inclusion of the studies, the studies were divided into two main categories which included curriculum-integrated interventions and stand-alone courses. Studies were classified as curriculum-integrated studies if no specific course or workshop was created as a separate intervention from the participants’ current curriculum. To be a curriculum-integrated study, the study must integrate concepts that aim to enhance communication skills into the existing clinical curriculum, either vertically or horizontally, that exists at the medical program. Any study that created an intervention and added it as a dedicated separate course was classified as stand-alone. The stand-alone courses were further stratified into four types of interventions. These interventions included Training Courses (workshops including instructions focusing on communication skills such as lectures, seminars, work in smaller groups and role play with tutors and other students), Drama Courses (simulation based approaches such as Forum Theater), Interactive Patient Learning Courses (such as learning through interactions with standardised or virtual patients) and Community-Based Learning Courses (combining learning experience with community service). As the data set of outcome measures was multi-dimensional, gathering information from different constructs based on measurements of different aspects of communication skills, normalization was used to enable data interpretation. These measurements included the Jefferson Scale of Physician Empathy (JSPE), the Calgary-Cambridge communication guide, various knowledge-based tests, self-assessments, faculty questionaries, and individual communication rating scales. Normalization allows the ability to extract knowledge from multi-dimensional data sets and plays a critical role in the development of artificial neural networks applicable in educational data mining [31], generating practical and applicable information from diverse data sets. The numerical outcomes of individual studies were normalized into a 0–1 scale using min-max normalization [31]. Given the minimal score and maximal score for each kind of assessment, the scores were transformed into a decimal between 0 and 1. After data normalization, we calculated the benefits from training for each study, which is the difference between normalized post-test and pre-test scores. After data was normalized, two-sample paired t-tests were used to compare the scores of male versus female students, including pre-test and post-test scores, and benefits from training (difference between post-test and pre-test scores). Normalized pre-test scores and post-test scores were also compared within each gender with paired t-test. Secondly, the different categories of interventions used in studies were analyzed based on available data (Training Course and Curriculum-Integrated). Sensitivity analysis was performed excluding studies with high or critical level of bias.

### Bias assessment

Bias assessment was performed with the I-ROBINS tool for non-randomized and RoB2 for randomized studies (L.B and A.M.) [32, 33].

## Results

### Search results

**Fig. 1:**
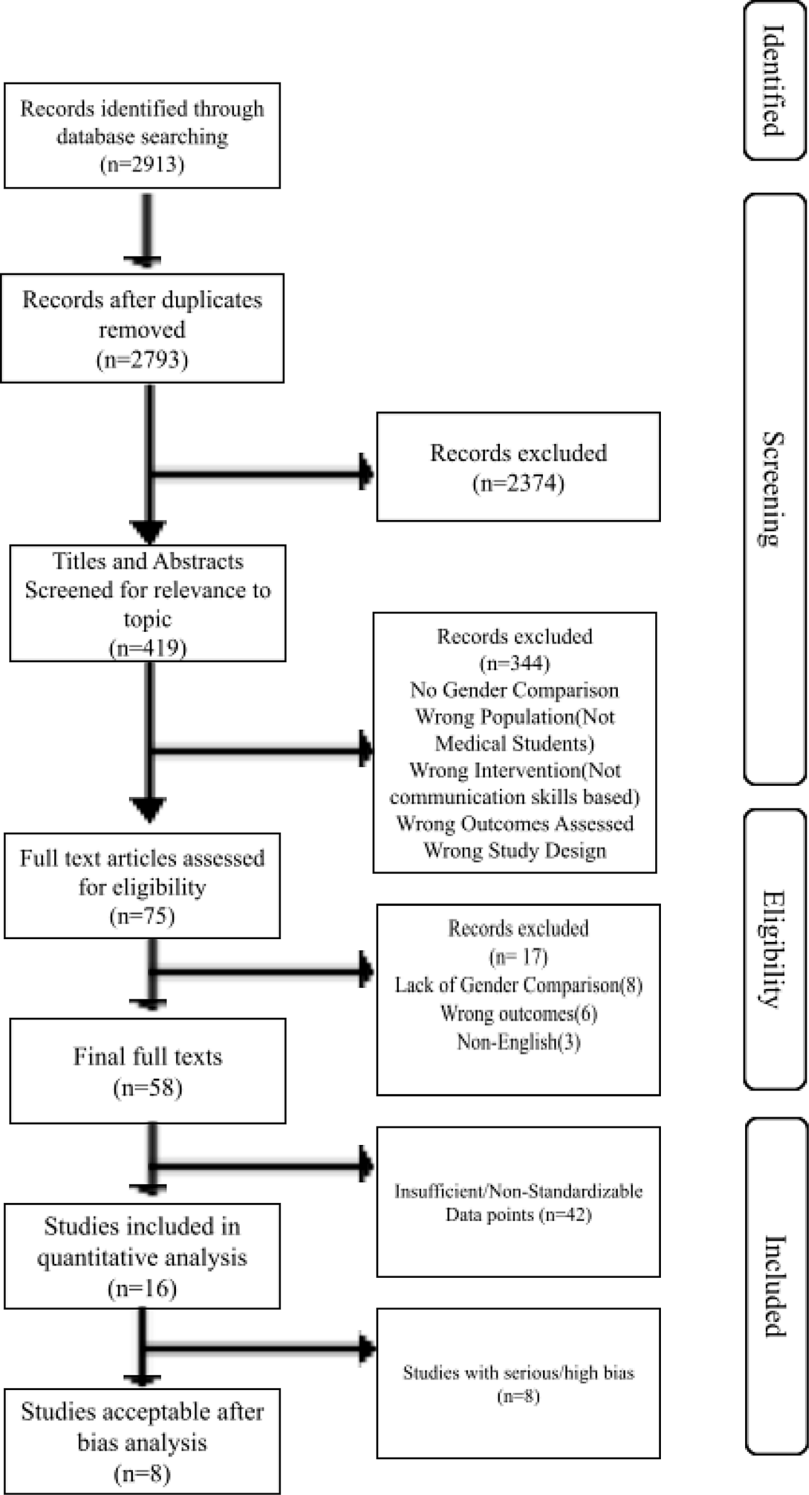
PRISMA flowchart depicting study selection methodology. PRISMA, preferred reporting items for systematic reviews

2913 articles were identified based on search terms (see Fig. 1). After duplicates were removed, 2793 articles were identified. 2374 were excluded at the title and abstract level if they did not describe a communication-based intervention or assessment for medical students, resulting in 419 articles proceeding to the full-text review. 344 articles were excluded in the initial round of full-text review if gender outcomes were not provided, improper study design was in place, the study measured wrong outcomes from Kirkpatrick’s hierarchy (level I or II), the intervention was not aimed to improve communication skills, or the intervention was not assessing medical students.

75 articles met the criteria for assessment and intervention and were assessed for final full-text review and discussed by five researchers involved in the study selection. Full-text articles were not available for 3 articles. 6 articles were excluded due to inadequate intervention or assessment. 8 articles were excluded due to a lack of comparison between genders, resulting in 58 articles included after a full-text review was completed [34–89]. 

### Intervention strategies

58 studies were included in this review, published between the years of 1991–2021,with a total of 7819 participants. The studies included a variety of educational interventions aiming to improve communication skills. 29 articles using Training Course-based interventions, 16 articles using Curriculum-Integrated Training, 2 articles using Drama Courses, 3 articles using Community-Based Learning Courses, and 8 articles using Interactive Patient Learning Courses were included in this review (Table 1). The studies included at this stage reported in their Methods section that pre and post-test assessment were performed and reported if male, female or all students improved after intervention. All articles reported post-intervention improved communication training for both male and female medical students. Most (69%) of the articles reported improved communication for men and women equally based on the statistical test used in the individual article. 28% of articles reported improved communication skills for women significantly more than men and 3% of articles reported improved communication skills for men significantly more than women.

**Table 1:** Stratification of fifty-eight articles which originally underwent full-text review by intervention category and reported results

Intervention categories	Reported results
Training Course (29)	Equal (22) Female (7) Male (0)
Curriculum-Integrated (16)	Equal (12) Female (4) Male (0)
Interactive Patient Learning Courses (8)	Equal (5) Female (2) Male (1)
Community-Based Learning Courses (3)	Equal (0) Female (2) Male (1)
Drama Course (2)	Equal (1) Female (1) Male (0)

Legend

Equal = no statistically significant difference between male and female students.

Female = female students obtained significantly better results than male students.

Male = male students obtained significantly better results than female students

### Assessment of the effectiveness of educational interventions in improving communication skills according to gender

Of the 58 articles that used adequate interventions and assessments to improve and evaluate intergender communication skills, a subgroup of 16 studies reported numerical outcomes for both pre-test and post-test scores (rather than only providing information who benefited from intervention) which were sufficient for data analysis, as described in the methods section. Included studies as summarized in Supplemental Table 2, with study design, number of participants, and measured outcome.

### Bias assessment

**Fig. 2:**
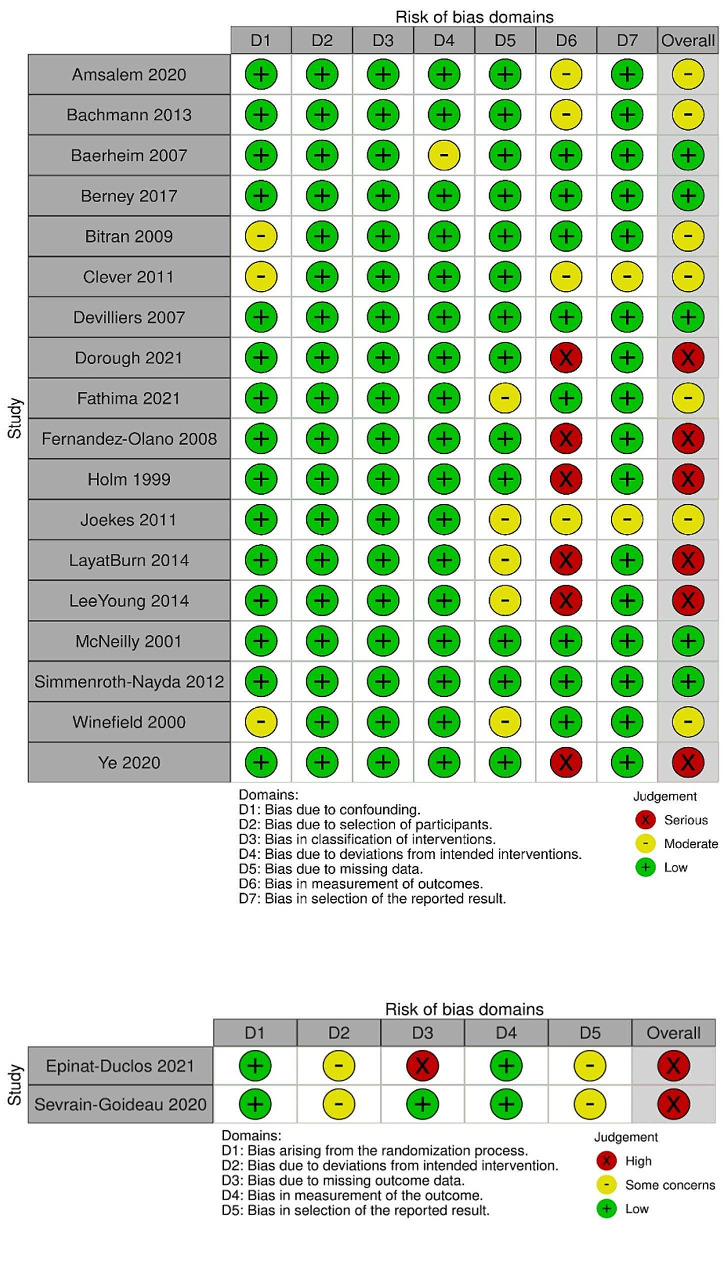
Bias assessment results of non-randomized studies with I-ROBBINS (top) and randomized studies with ROB-2 (bottom)

Bias assessment was performed only for the subgroup of 16 studies included in the statistical analysis (Fig. 2). 14 of the studies were non-randomized and two were randomized. The overall risk of bias was moderate. For the non-randomized studies, the domain where severe levels of bias were found was the measurement of outcomes. For the randomized studies, high risk of bias was found in the domain of risk for deviations from intended interventions.

### Intervention program outcomes

**Fig. 3:**
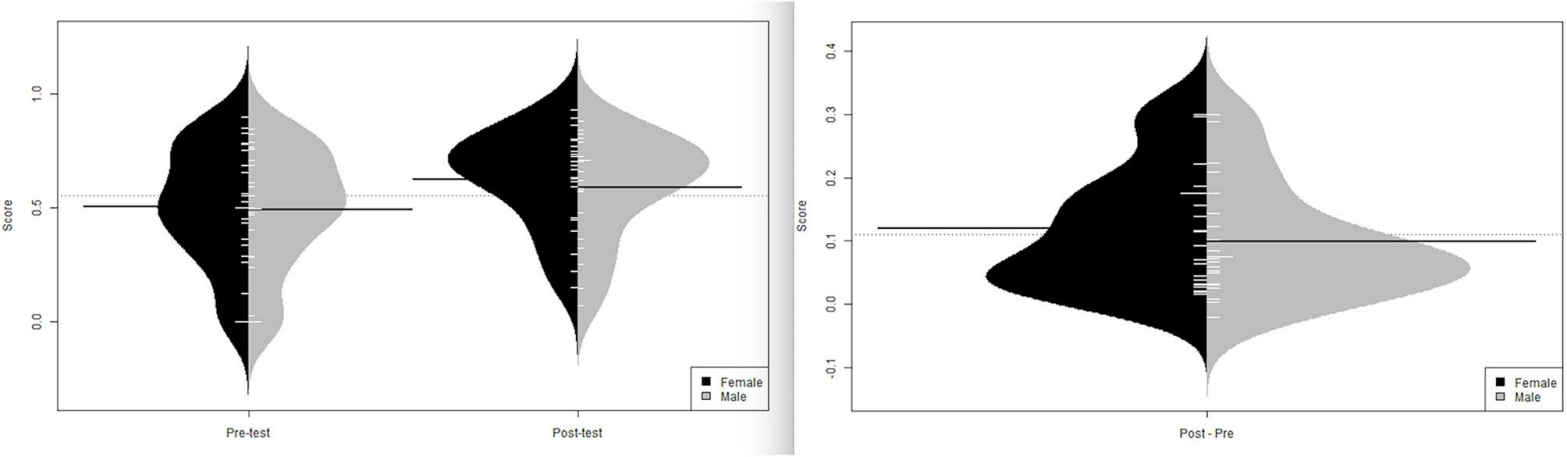
Bean plots of pre-intervention and post-intervention scores (left) and the difference between them (right)

The subgroup of 16 studies (7819 participants) was analyzed to determine differences in the effectiveness of improving the communication skills of both genders. Using two sample paired t-tests on min-max normalized scores to compare male scores versus female scores, and pre-test versus post-test generated the following results. For the pre-test scores, there was no significant difference between the population means of male (0.490) and female (0.504) students (*p* = 0.177, t=-1.397, df = 21). However, for the post-test scores, the population mean of male students (0.589) was significantly lower than that of female (0.624) students (*p* = 0.006, t=-2.738, df = 21). For both male and female students, the population mean of post-test scores was significantly greater than that of pre-test scores, with *p*-values < 0.001 (males and females t=-5.183, df = 21 and t=-5.870, df = 21, respectively) (Fig. 3). For the difference between post-test and pre-test scores (benefits from training), overall, there is no significant difference between the population means of male (0.099) and female (0.120) students (*p* = 0.154, t=-1.478, df = 21) (Fig. 3).

Categorized interventions were also analyzed using paired t-tests on normalized scores. In the Curriculum-Integrated category of intervention, there is no significant difference between the population means of male students’ benefits (0.0119) and female students’ benefits (0.135) (*p* = 0.528, t=-0.655, df = 10). However, in the Training Course category of intervention, the population mean of male medical students’ benefits (0.062) was significantly less than that of female medical students’ benefits (0.097) at the 5% significance level (*p* = 0.041, t=-2.094, df = 6).

Sensitivity analysis was performed after excluding eight studies with high or critical levels of bias (see Fig. 2). For the post-test scores, the population mean of male students normalized scores were still significantly lower than the female students, however only significant at 5% significance level (pre-sensitivity analysis *p*-value was 0.006, post-sensitivity analysis *p*-value was 0.029). All the comparisons about Training courses, such as comparing effectiveness of Training courses vs. Curriculum-integrated, and comparing the population means between male and female students in the Training Course category of no longer possible as sample size in the Training courses category was too small, as only three studies with this course design could have been included. All other results were unchanged. 

## Discussion

In this review, we have included studies describing outcomes of educational interventions, divided into non-integrated Training Courses, Curriculum-Integrated communication skills teaching, and Interactive Patient Learning Courses, Community-Based Learning Courses, and Drama courses. Overall, all studies found that both male and female medical students improved their communication skills when compared to pre-test values. The majority of the 58 included studies reported no significant differences between groups of male and female students post-intervention, 28% reported that female students performed significantly better than males, and only 2 studies showed that male students improve their communication skills significantly more than female students.

In the analysis of a subgroup of 16 studies with sufficient reported data, we found that there was no difference in pre-test scores of male versus female students. However, post-test scores showed that while both male and female students improved, female students obtained higher scores. When the design of educational intervention was considered, we found that curriculum-integrated courses benefited both male and female students. Weak support at a 5% significance level was found that male students might benefit less from non-integrated training courses compared to communication skills education integrated into the curriculum.

Stand-alone, non-integrated training courses and curriculum-integrated interventions are the two most frequently used strategies to teach communication skills. In a systematic scoping review of communication skills teaching in medical schools, integrated design of communication skills teaching into a formal curriculum, with appropriate integration both horizontally and vertically, has been identified as one of the enablers fostering learning [13]. Several primary studies comparing integrated and stand-alone curriculum designs in communication skills showed superior outcomes of integrated intervention [90, 91]. The potential risk associated with stand-alone communication skills courses is that of a ‘silo effect’, where students struggle with generalizing the knowledge [91]. This risk might be lower for female students, who in general seem to acquire communication skills more easily.

Gender has been implicated as a strong factor in shaping medical education through teaching focused on male diagnosticians and scientists, that male physicians occupy the majority of academic and leadership positions, the presence of sexual harassment, and gender segregation in specialties [92]. Viewed through the lens of feminist theory, attempts to introduce gender equality are seen as largely leading to the assimilation of women into male-dominated environments rather than transforming them, maintaining a disadvantaged position of women in medicine [93]. Both formal and hidden curricula are likely to affect medical students in a gendered way. In a systematic review, the hidden curriculum was found to have negative connotations, implying a conflict with the formal curriculum [94]. Far fewer studies depicted the hidden curriculum as positive for medical students. The concepts about how the hidden curriculum affects medical students resonate with theories of situated learning and communities of practice, where professional learning occurs through the learner’s participation in the community and incorporating its sociocultural practices [94, 95]. When exploring the interplay between gender and hidden curriculum, a study analyzing the content of communication platforms used by students and faculty found that both teachers and students contributed to a heterosexual masculine culture and sexism, resulting in male students seen by the faculty as their potential successors [96]. On the other hand, students of both genders experienced gendered ‘othering’ in the analysis of the content of e-portfolios on gender and health, though through different pedagogical strategies, with male students complaining of a lack of support when trying to acquire genealogical examination skills [97]. We could not find any studies reporting of any other specific difficulties for male medical students or male students in higher education in general [98, 99].

It is also largely unknown if male medical students or their teachers are aware of a large body of literature detailing gender differences in communication skills. However, awareness of underperforming compared to women could potentially have negative effects on the learning of male students. A study examining school education found that boys are generally aware of stereotype threat (not performing as well as girls in domains such as reading) and consequently motivated to overcome their group’s negative depiction but the awareness of stereotype threat did not enable them to improve their performance [100]. Further investigation is required to understand how and why current communication skills seem to favor female medical students as seen in better post test results in this study. Potential factors could be at play, including the fact that stand-alone courses may more likely be taught by faculty with a special interest in communication skills rather than regular faculty. This discrepancy could potentially favor students who are actively engaged in the subject and acquire communication skills with ease, playing to the strengths of the strongest students [101].

Another possible explanation could be that male students respond to a higher extent to learning with rather than learning about [102]. While not explored within the context of medical education, in language learning, boys are affected to a higher extent by unsupportive peer groups and teachers [103].

The unfulfilled potential of male students coexists with increasing expectations that the physician will possess strong communication skills and a caring side, which were historically expected of female physicians. The medical field is experiencing a shift with more women than men entering the profession, possibly accelerating this cultural change [104]. This should motivate the community involved in medical education to address these challenges, as there is a risk that medical education favors female students and thus fails to prepare men adequately.

### Implications for practice

Integrated communication skills courses benefit both male and female medical students. While not detrimental for female medical students, stand-alone training courses in communication skills might lead to worse performance for male medical students. When designing medical curricula, communication skills ought to be taught, at least partially, in a curriculum-integrated manner, until more is known on the subject, to avoid widening the gender gap in communication skills.

### Future research

A Delphi study on outcomes recommended for the assessment of communication skills, involving researchers and medical educators would inform future study designs and enable meaningful comparisons [105]. Studies exploring perceptions of male medical students and their teachers through a qualitative paradigm could shed light on factors effecting learning of communications skills for this group.

### Strengths and weaknesses

Most of the included studies are non-randomized, with opportunistic designs and outcomes at Kirkpatrick levels III-IV. Although no language limitations were imposed, most studies originated in Europe and USA, limiting the transferability of the results. This review focuses on binary gender (male-female), based on the methodological limitations of including studies, where it was not always possible to ascertain if the participants identified themselves as belonging to one of the binary genders. This study does not consider other intersectional factors, as including intersectionality would limit the number of studies which could be included.

## Conclusion

The results suggests that female medical students perform better in various aspects of communication skills than male students after educational interventions, even if there is no difference in benefits of interventions for both genders. There is weak support that curriculum-integrated communication skills teaching is more beneficial for male medical students as compared to stand-alone courses.

### Electronic supplementary material

Below is the link to the electronic supplementary material.

Supplementary Material 1

Supplementary Material 2

## Data Availability

All data generated or analyzed during this study are included in this published article [and its supplementary information files]. Further data is available upon request of corresponding author.
