# Supplementary material for: Do educational interventions reduce the gender gap in communication skills?- a systematic review
Source: BMC Med Educ. 2024 Jul 31;24:827. doi: 10.1186/s12909-024-05773-9 (PMC11293108; doi:10.1186/s12909-024-05773-9)
Supplement: Supplementary file 1 — Supplementary Material 1 [file 12909_2024_5773_MOESM1_ESM.docx]

**SUPPLEMENTAL MATERIAL**

**Table 1. Complete search strategy used for article selection.**

| (“students, medical”[MeSH Terms] OR (“students”[All Fields] AND “medical”[All Fields]) OR “medical students”[All Fields] OR (“medical”[All Fields] AND “students”[All Fields]) OR ("education, medical"[MeSH Terms] OR ("education"[All Fields] AND "medical"[All Fields]) OR "medical education"[All Fields] OR ("medical"[All Fields] AND "education"[All  Fields])) OR ("schools, medical"[MeSH Terms] OR ("schools"[All Fields] AND "medical"[All Fields]) OR "medical schools "[All Fields] OR (“medical [All Fields] AND "school"[All Fields]) OR "medical school"(All Fields])) AND (("communicate "[All Fields] OR "communicated"[All Fields] OR "communicates"[AIl Fields] OR "communicating"[All Fields] OR "communication"[MeSH Terms] OR "communication"[All Fields] OR "communications"[All Fields] OR "communicative"[All Fields] OR "communicational"[All Fields] OR "communicatively"[All Fields] OR "communicativeness"[All Fields] OR "communicator"[All Fields] OR "communicator s"[All Fields] OR"communicators"[All Fields]) AND ("skill"[All Fields] OR "skilled"[All Fields] OR "skillful"[All Fields] OR "skillfulness"[All Fields] OR "skills"[All Fields])) AND ("education"[MeSH Subheading] OR "education"[All Fields] OR "teaching”[All Fields] OR "teaching"[MeSH Terms] OR "teaches"[All Fields] OR "teach"[All Fields] OR "teachings"[All Fields] OR "teaching s"[All Fields] OR ("learning"[MeSH Terms] OR "learning"[All Fields] OR "learn"[All Fields] OR "learned"[All Fields] OR "learning s"[All Fields] OR "learnings"[All Fields] OR "learns"[All Fields])) AND ("gender identity”[MeSH Terms] OR ("gender"[All Fields] AND "identity”[All Fields]) OR "gender identity"[All Fields] OR "gendered"[All Fields] OR "gender s"[All Fields] OR "gendering"[All Fields] OR "genderized"[All Fields] OR "genders"[All Fields] OR "sex"[MeSH Terms] OR "sex"[All Fields] OR "gender"[All Fields]) |
| --- |

**Table 2. Summary of studies sufficient for data analysis by study design, classification, number of participants, Kirkpatrick level of hierarchy, and measured outcome.**

| **Study** | **Study Design** | **Study Classification** | **Number of Participants** | **Kirkpatrick Level of Assessment** | **Measured Outcome** |
| --- | --- | --- | --- | --- | --- |
| Bachmann 2013 | non-randomized | Patient Interactive Experience | 80 | III | Self-assessment, OSCE |
| Bitran 2009 | non-randomized | Training Course | 173 | III | Writing process rubric |
| DeVilliers 2007 | non-randomized | Training Course | 161 | III | Calgary-Cambridge communication guide |
| Dorough 2021 | non-randomized | Training Course | 116 | II | Jefferson Scale of Physician Empathy (JSPE) |
| Epinat-Duclos 2021 | randomized | Training Course | 312 | II | Jefferson Scale of Physician Empathy (JSPE) |
| Fathima 2021 | non-randomized | Community-Based Project | 142 | II | Knowledge-based questionnaire |
| Fernandez-Olano 2008 | non-randomized | Training Course | 203 | II | Jefferson Scale of Physician Empathy (JSPE) |
| Holm 1999 | non-randomized | Curriculum-Integrated | 240 | II | Affect Reading Scale |
| Joekes 2011 | non-randomized | Curriculum-Integrated | 82 | III | Evans Interview Rating Scale |
| LayatBurn 2014 | non-randomized | Training Course | 225 | II | Self-assessment |
| LeeYoung 2014 | non-randomized | Curriculum-Integrated | 111 | III | Faculty-rated questionnaire |
| McNeilly 2001 | non-randomized | Curriculum-Integrated | 72 | II | Knowledge-based questionnaire |
| Sevrain-Goideau 2020 | randomized | Acting Course | 488 | II | Jefferson Scale of Physician Empathy (JSPE), self-assessment |
| Simmenroth-Nayda 2012 | non-randomized | Training Course | 32 | III | Calgary-Cambridge communication guide |
| Winefield 2000 | non-randomized | Training Course | 115 | II | Empathy likert scale |
| Ye 2020 | non-randomized | Curriculum-Integrated | 257 | II | Jefferson Scale of Physician Empathy (JSPE) |
