# Supplementary material for: Do educational interventions reduce the gender gap in communication skills?- a systematic review
Source: BMC Med Educ. 2024 Jul 31;24:827. doi: 10.1186/s12909-024-05773-9 (PMC11293108; doi:10.1186/s12909-024-05773-9)
Supplement: Supplementary file 2 — Supplementary Material 2 [file 12909_2024_5773_MOESM2_ESM.docx]

**Table 2. Summary of studies included for data analysis by study design, classification, number of participants, Kirkpatrick level of hierarchy, and measured outcome.**

| **Study** | **Study Classification** | **Number of Participants** | **Measured Outcome** |
| --- | --- | --- | --- |
| Amsalem 2020 | Interactive Patient Learning Course | 42 | Four Habiits Coding Scale (4-HCS) |
| Austin 2005 | Curriculum-Integrated | 156 | Emotional Intelligence (EI) scale |
| Bachmann 2013 | Interactive Patient Learning Course | 80 | Self-assessment, OSCE |
| Baerheim 2007 | Curriculum-Integrated | 1801 | Knowledge-based questionnaire |
| Berney 2017 | Interactive Patient Learning Course | 236 | Calgary-Cambridge communication guide |
| Bitran 2009 | Training Course | 173 | Writing process rubric |
| Bonnaud-Antignac 2010 | Training Course | 108 | Self-assessment |
| Burn 2014 | Training Course | 225 | Self-assessment |
| Christopher 2021 | Community-Based Learning Course | 11 | Clinical Learning Environment Comparison Survey |
| Chur-Hansen 2001 | Interactive Patient Learning Course | 127 | Australian Tertiary English Screening Test, self-assessment |
| Clever 2011 | Interactive Patient Learning Course | 237 | Self-assessment |
| Cohen-Schotanus 2008 | Curriculum-Integrated | 344 | Self-assessment |
| Courteille 2014 | Interactive Patient Learning Course |  | Self-assessment |
| DeVilliers 2007 | Training Course | 161 | Calgary-Cambridge communication guide |
| Dorough 2021 | Training Course | 116 | Jefferson Scale of Physician Empathy (JSPE) |
| Epinat-Duclos 2021 | Training Course | 312 | Jefferson Scale of Physician Empathy (JSPE) |
| Fathima 2021 | Community-Based Learning Course | 142 | Knowledge-based questionnaire |
| Fernandez-Olano 2008 | Training Course | 203 | Jefferson Scale of Physician Empathy (JSPE) |
| Franco 2020 | Training Course | 69 | Knowledge-based questionnaire and qualitative analysis |
| Grossman 2021 | Drama Course | 188 | OSCE, patient satisfaction questionnaire |
| Hashim 2013 | Interactive Patient Learning Course | 45 | Self-assessment |
| Holm 1999 | Curriculum-Integrated | 240 | Affect Reading Scale |
| Iqbal 2015 | Training Course | 52 | Calgary-Cambridge communication guide |
| Joekes 2011 | Curriculum-Integrated | 82 | Evans Interview Rating Scale |
| Keen 2015 | Training Course | 211 | Patient centered observation form |
| Kim 2020 | Training Course | 45 | Empathy self-assessment |
| Lee 2014 | Curriculum-Integrated | 111 | Faculty-rated questionnaire |
| Lie 2010 | Curriculum-Integrated | 192 | Patient-physician interaction scale |
| Lim 2011 | Training Course | 149 | OSCE, Jefferson Scale of Physician Empathy (JSPE) |
| Lim 2013 | Training Course | 72 | OSCE, Jefferson Scale of Physician Empathy (JSPE) |
| Lim 2016 | Curriculum-Integrated | 79 | OSCE, Jefferson Scale of Physician Empathy (JSPE) |
| Luttenberger 2014 | Training Course | 182 | Self-assessment |
| Madjar 2015 | Training Course | 151 | Self-assessment |
| Marteau 1991 | Training Course | 88 | Attitude-based questionnaire |
| McNeilly 2001 | Curriculum-Integrated | 72 | Knowledge-based questionnaire |
| Nayak 2021 | Training Course | 300 | Course effectiveness questionnaire |
| Price 2008 | Training Course | 120 | OSCE |
| Przymuszala 2021 | Curriculum-Integrated | 126 | Self-assessment |
| Qureshi 2020 | Curriculum-Integrated | 80 | OSCE with Liverpool Communication Skills Assessment Scale |
| Rauch 2021 | Training Course | 306 | OSCE, self-assessment |
| Sahu 2018 | Curriculum-Integrated | 195 | Attitude-based questionnaire |
| Saldert 2016 | Training Course | 59 | Knowledge- and attitude-based questionnaire |
| Samuels 2021 | Training Course | 137 | Self-assessment |
| San-Martin 2017 | Training Course | 165 | Jefferson Scale of Physician Empathy (JSPE) |
| Schmitz 2018 | Training Course | 67 | OSCE |
| Sevrain-Goideau 2020 | Drama Course | 488 | Jefferson Scale of Physician Empathy (JSPE), self-assessment |
| Sezer 2019 | Curriculum-Integrated | 92 | Knowledge- and attitude-based questionnaire |
| Shapiro 2006 | Training Course | 92 | Qualitative analysis (essays) |
| Snow 2016 | Interactive Patient Learning Course | 88 | OSCE |
| Taveira-Gomes 2016 | Curriculum-Integrated | 255 | Standardized Patient (SP) interview |
| Simmenroth-Nayda 2012 | Training Course | 32 | Calgary-Cambridge communication guide |
| Tiuraniemi 2011 | Training Course | 107 | Self-assessment |
| vonLengerke 2011 | Training Course | 267 | Self-assessment |
| Wee 2010 | Community-Based Learning Course | 576 | Attitude-based questionnaire, self-assessment |
| Winefield 2000 | Training Course | 115 | Empathy likert scale |
| Windish 2005 | Training Course | 120 | OSCE |
| Ye 2020 | Curriculum-Integrated | 257 | Jefferson Scale of Physician Empathy (JSPE) |
| Yu 2016 | Training Course | 82 | Micro- and subtle expression training tools (METT, SETT) |
